# Public Health Impact of Potential Infant MenACWY Vaccination Strategies in Spain

**DOI:** 10.3390/vaccines13060642

**Published:** 2025-06-13

**Authors:** Katharina Schley, Jamie Findlow, Carlos Molina, Shannon M. Sullivan, Eszter Tichy

**Affiliations:** 1 Global Access and Value, Pfizer Pharma GmbH, 10117 Berlin, Germany; 2 Pfizer Global Medical Affairs, Vaccines and Antivirals, Pfizer Ltd., Tadworth KT20 7NS, UK; 3 Medical Department, Pfizer S.L.U., 28108 Madrid, Spain; 4 PPD Evidera Health Economics & Market Access, Thermo Fisher Scientific, 94853 Ivry-sur-Seine Cedex, France; 5 PPD Evidera Health Economics & Market Access, Thermo Fisher Scientific, H-1113 Budapest, Hungary

**Keywords:** immunization, invasive meningococcal disease, meningococcal vaccine, public health, Spain, vaccine

## Abstract

**Background:** The Spanish Interterritorial Council of the National Health System (a central government body) currently recommends vaccination against meningococcal serogroup C (MenC) at 4 and 12 months of age for prevention of invasive meningococcal disease (IMD). The Advisory Committee on Vaccines of the Spanish Association of Pediatrics (a professional medical association) and numerous Spanish regional bodies instead recommend quadrivalent vaccination against serogroups A, C, W, and Y (MenACWY) at 4 and 12 months of age. The central government and Spanish Association of Pediatrics also recommend MenACWY vaccination at 12 years of age. This study assessed the potential public health effects of replacing the MenC vaccination schedule with different MenACWY vaccination schedules in infants. **Methods:** Here, a static multi-cohort population model was used to evaluate potential effects on public health of IMD due to meningococcal serogroups C/W/Y, comparing MenC infant vaccination (reference strategy) against four different strategies including quadrivalent tetanus toxoid conjugate vaccine (MenACWY-TT; Nimenrix^®^, Pfizer Europe MA EEIG, Brussels, Belgium) infant vaccination; all strategies included MenACWY-TT vaccination at 12 years of age. **Results:** The most effective strategy for infant vaccination was MenACWY-TT at 2, 4, and 12 months, preventing an estimated additional 103 IMD cases, 17 deaths, and 41 cases with long-term sequelae (LTS) versus the reference strategy in the base-case IMD incidence scenario. When strategies included a two-dose infant schedule, the earlier the infant MenACWY-TT vaccine was administered, the more additional cases, deaths, and cases with LTS were prevented (base-case and high-incidence scenarios). **Conclusions:** This analysis supports implementation of MenACWY-TT as a replacement for MenC vaccination.

## 1. Introduction

Invasive meningococcal disease (IMD) is a rare but serious bacterial infection caused by *Neisseria meningitidis* and is associated with high mortality and substantial long-term sequelae (LTS) among survivors [1,2]. Initial symptoms are typically nonspecific, and the disease can rapidly progress and become fatal within hours [3]. A recent meta-analysis of studies that reported clinical epidemiology of IMD estimated an overall case fatality rate of 8.3% (derived from 29 studies, with data from many countries/regions) [2]. Among survivors, more than a third experience varied debilitating LTS, including limb amputation, hearing loss, cognitive impairment, and emotional and behavioral difficulties [1,4]. LTS due to IMD also adversely affect the health-related quality of life of survivors, their families, and caregivers [4].

The epidemiology of IMD is unpredictable because of regional shifts in predominant disease-causing serogroups over time and occurrences of sporadic outbreaks [3,5]. A total of 12 meningococcal serogroups have been determined; surveillance data indicate that the serogroups B, C, W, and Y (MenB, MenC, MenW, and MenY, respectively) cause almost all recent IMD cases in Europe, and that serogroup A (MenA) contributes to IMD in other regions [1,3]. The incidence of IMD also varies with age. Infants and young children are at highest risk for IMD, with many countries experiencing a secondary peak in incidence in adolescents/young adults [3,5]. The serogroup distribution of IMD case isolates has evolved dramatically across Europe in the last decade; data from the European Surveillance System (TESSy) indicated that the prevalence of MenW increased from 1.7% in 2008 to 16.0% in 2017, making it the second-most prevalent serogroup; over the same period the prevalence of MenY increased from 3.0% to 10.9%; in contrast, the prevalence of MenC remained similar, i.e., 14.4% in 2008 and 15.3% in 2017; and the prevalence of MenB decreased from 71.5% to 48.0% [6]. This increase in the prevalence of MenW and MenY has been particularly marked among older adults, who often develop atypical symptoms, including gastrointestinal and respiratory symptoms, resulting in delayed diagnosis or misdiagnosis, and potentially rapid disease progression and death, highlighting the importance of protecting against these serogroups [3,7,8]. In Spain, as in many countries across the world, IMD incidence decreased during the COVID-19 pandemic (2020 and 2021), likely due to implementation of lockdowns, social distancing, and containment measures; there were 64 IMD cases in 2020/2021 compared with 390 cases in 2018/2019 and 270 cases in 2019/2020 [9,10,11,12]. However, since the end of the pandemic, IMD cases in Spain have been increasing again [13]. In 2021/2022, 108 confirmed IMD cases were reported; recent provisional data indicate there were 248 (131 MenB, 4 MenC, 29 MenW, and 23 MenY) and 311 (1 MenA, 135 MenB, 5 MenC, 25 MenW, and 14 MenY) confirmed cases in 2023 and 2024 (through Week 50), respectively [13,14].

Vaccination is an effective measure against IMD, and many countries have recommendations for meningococcal vaccines in their national immunization programs (NIPs) [15]. Recently, in response to the increase in MenW and MenY IMD, many countries incorporated quadrivalent conjugate vaccines against serogroups A, C, W, and Y (MenACWY) into their NIPs. Real-world studies have shown that the introduction of MenACWY vaccines into NIPs has been highly effective at reducing IMD incidence among vaccine-eligible age groups (typically adolescents) and can also provide indirect protection (i.e., herd protection) in non-vaccine-eligible age groups [16]. In Spain, a nationwide schedule for recommended vaccinations has not been implemented; the Spanish Interterritorial Council of the National Health System (a central government body) recommends MenC vaccination at 4 and 12 months of age, and MenACWY vaccination at 12 years of age [17]; however, the regional governments of autonomous communities of Spain may independently implement vaccination schedules according to need, and may or may not adhere to the Spanish Interterritorial Council of the National Health System recommendations. In 2024, to better protect children aged 1 to 12 years of age against IMD, the vaccination schedule issued by the Advisory Committee on Vaccines of the Spanish Association of Pediatrics (a national professional medical association) was updated to recommend replacement of MenC vaccination at 4 and 12 months of age with the quadrivalent tetanus toxoid conjugate vaccine (MenACWY-TT; Nimenrix^®^, Pfizer Europe MA EEIG, Brussels, Belgium) at 4 and 12 months of age, whilst maintaining quadrivalent MenACWY vaccination at 12 years of age [18]. This recommendation has been adopted by several Spanish regions (Andalucía, Galicia, Castilla y León [19,20,21], and Extremadura) ahead of any potential change to the national recommendations. Therefore, we developed a mathematical model to assess the potential public health impact in Spain of replacing infant MenC vaccination with various infant schedules incorporating MenACWY-TT; all schedules also included MenACWY-TT vaccination at 12 years of age.

## 2. Methods

**Figure 1:**
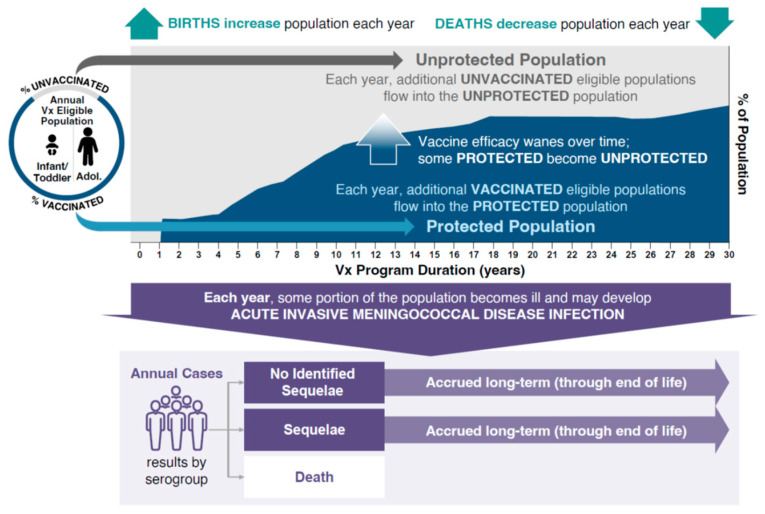
Static-population cohort model. Adol, adolescents; Vx, vaccination.

The burden of IMD and associated outcomes were analyzed using a static multi-cohort population model (Microsoft Excel^®^, version 2503; Microsoft Corporation, Redmond, WA, USA). The model evaluated the public health impact of different vaccination strategies while simulating the clinical course of IMD in the Spanish population (Figure 1). The model estimated the size of the population every year and at every age based on the population in Spain. The number of serogroup C, W, and Y (MenC/W/Y) cases in each age group was calculated with case numbers being reduced based on the effectiveness of the vaccination strategy entered into the model. To simulate the potential public health impact of IMD due to MenC/W/Y, the model used a base-case incidence rate for 11 age groups using averages of European Centre for Disease Prevention and Control incidence data from 1999 to 2006 (MenC) and from 2016 to 2019 (MenY and MenW) stratified by age group to account for differences in disease epidemiology and the effect of the MenC immunization program (Table 1). MenC immunization was introduced in 2000 following an increased burden of MenC disease due to an outbreak. Using the average disease incidence from 1999 to 2006 approximates a pre-vaccination incidence without overestimating the pre-vaccination incidence using only data from 1999–2000, which was driven by a disease outbreak. By using historic incidence rates, potential double counting is avoided that could occur if vaccine effectiveness was applied to incidence estimates that were already decreased due to the effects of an immunization program. Examination of yearly incidence data revealed peaks in incidence that were used to inform hypothetical high-incidence scenarios for MenC (used average incidence during 1999–2000 peak), and MenW and MenY (used average incidence during the 2018–2019 peak).

This model allowed for comparison of any two vaccination strategies. Four vaccination strategies were analyzed using the model, focusing on the adoption of MenACWY-TT vaccination to replace MenC vaccination in infants (the reference strategy). All vaccination strategies incorporated a MenACWY-TT dose at 12 years of age. A single vaccine effectiveness was assumed for each vaccine class and dose (Table 2). Vaccine effectiveness was derived from published data for the MenACWY-TT and MenC vaccines [22,23,24,25]. The model assumed annual waning of vaccine effectiveness based on exponential decay, dependent on the age at which the vaccine was given: infant, 22.12%; adolescent, 5.85%. These assumptions were aligned with a previous model that assessed the impact of meningococcal vaccination on public health in the UK [26], and were based on observations of vaccine persistence/decay derived from immunogenicity and human complement serum bactericidal assay titer data with MenACWY-TT [27,28,29]. IMD due to MenB was out of scope for this analysis. Meningococcal serogroup A incidence in Spain is very low (0 cases in 2023 and provisionally 1 case in 2024 through Week 50) and was therefore not considered [14,30]. Based on vaccine coverage data from the Spanish Ministry of Health, the modeled inputs for vaccine uptake were 97% for the first dose and 95% for any second dose administered before 12 months of age, 93% for doses administered at 12 months of age, and 90% for doses administered at 12 years of age [31,32]. The model structure analyzed the effects of each vaccination strategy by adopting a 30-year time horizon in which new population cohorts entered the model for 30 years; individuals in the cohort were followed until death, and impacts on the public health burden of IMD (i.e., numbers of cases, deaths, and cases with LTS) were estimated (Figure 1). Based on real-world evidence following the introduction of MenACWY-TT into adolescent immunization programs in the Netherlands, Chile, and Australia, where IMD incidence was reduced as a result of indirect protection by 45–53% in age groups ineligible for vaccination, incidence rates in the unvaccinated population in the current model were reduced by 50% after the introduction of MenACWY-TT to account for the effects of herd protection [22].

## 3. Results

All modeled vaccination strategies that used MenACWY-TT to replace the reference vaccination strategy’s MenC infant doses, while maintaining the MenACWY-TT vaccination at 12 years of age, were associated with substantial estimated reductions in cases and deaths due to IMD (Table 3). The substitution of MenACWY-TT in infants resulted in an estimated additional reduction of 20 to 103 cases of IMD due to MenC/W/Y in the 30-year time horizon versus the reference strategy, with greater numbers of averted cases associated with an earlier age of administration of the primary dose. The modeled infant vaccination schedule of three doses of MenACWY-TT at 2, 4, and 12 months of age was associated with 103 fewer cases and 17 fewer deaths caused by IMD due to MenC/W/Y than the reference strategy. Administration of two doses of MenACWY-TT at 4 and 12 months of age, instead of the two doses of MenC at 4 and 12 months per the reference strategy, prevented an additional 60 cases and 11 deaths due to MenC/W/Y; vaccination with MenACWY-TT at 3 and 12 months of age prevented an additional 80 cases and 14 deaths due to MenC/W/Y. Delaying primary MenACWY-TT vaccination until 6 months of age (MenACWY-TT at 6 and 12 months) prevented an additional 20 cases and four deaths compared with the reference strategy. The prevention of sequelae followed a similar pattern, with reductions in the number of cases with LTS due to MenC/W/Y ranging from 7 to 41 when MenACWY-TT replaced MenC infant vaccinations. The greatest reduction in cases with LTS was predicted with the three-dose MenACWY-TT infant schedule at 2, 4, and 12 months of age; replacing MenC at 4 and 12 months of age with MenACWY-TT at 4 and 12 months resulted in a reduction of 24 cases with LTS.

To understand the effects of the vaccination strategies during times of high IMD incidence, several hypothetical high-incidence scenarios for MenC, MenW, and MenY were modeled. Across all high-incidence scenarios, substitution of MenC vaccination for MenACWY-TT vaccination in infants was associated with substantial reductions in estimated numbers of IMD cases, deaths, and cases with LTS, with the exception of the strategy in which the first infant MenACWY-TT dose was administered at 6 months (Table 4). Although there were benefits with this strategy in the high MenW and high MenY scenarios, in the high MenC scenario an additional 24, 2, and 11 cases, deaths, and cases with LTS were predicted versus the reference strategy, respectively. Model estimates indicated that the infant vaccination strategy of MenACWY-TT at 2, 4, and 12 months of age had the greatest impact across all high-incidence scenarios, resulting in the prevention of an additional 168 cases, 28 deaths, and 67 cases with LTS in the high MenC/W/Y incidence scenario versus the reference strategy; in the individual high MenC, MenW, and MenY incidence scenarios, this strategy prevented an estimated 112 to 133 additional cases, 18 to 23 deaths, and 45 to 53 cases with LTS. For the two-dose infant vaccination schedules in which the infant dose was administered at 3 or 4 months, the model estimated prevention of 124 and 86 additional cases, respectively, 22 and 16 deaths, and 49 and 34 cases with LTS in the high MenC/W/Y incidence scenario versus the reference strategy; and prevention of between 50 to 109 additional cases, 9 to 20 deaths, and 20 to 43 cases with LTS in the high MenC, MenW, and MenY incidence scenarios.

## 4. Discussion

The Spanish Interterritorial Council of the National Health System currently recommends a MenC vaccine at 2 and 4 months of age; however, the Advisory Committee on Vaccines of the Spanish Association of Pediatrics recently recommended replacing the infant MenC vaccinations with MenACWY-TT, citing greater protection against IMD in children between 1 and 12 years of age [17,18]. To better understand the public health impact in Spain of switching the infant MenC vaccine to MenACWY-TT, the potential benefits of replacing the MenC vaccination schedule with four different MenACWY-TT vaccination schedules were evaluated with a static multi-cohort population model.

Under the base (standard-incidence) scenario in this study, all four of the infant schedules—MenACWY-TT vaccination + MenACWY-TT vaccine at 12 years of age—prevented additional IMD cases, deaths, and cases with LTS compared with the reference two-dose infant MenC + MenACWY-TT vaccine at 12 years of age schedule. According to the model, the most effective strategy for protecting against IMD cases, IMD-related deaths, and cases with LTS was the three-dose infant MenACWY-TT schedule administered at 2, 4, and 12 months, with a fourth dose at 12 years; this prevented an additional 103 IMD cases, 17 deaths, and 41 cases with LTS. This MenACWY-TT schedule was also the most protective strategy under the hypothetical high MenC, MenW, and MenY incidence scenarios, preventing an additional 128, 133, and 112 IMD cases, respectively. Interestingly, under the high MenC incidence scenario, all of the infant MenACWY-TT vaccination strategies, except for the schedule with MenACWT-TT administered at 6 and 12 months of age with a further dose at 12 years, provided additional protection over the reference strategy.

When comparing the two-dose infant MenACWY-TT strategies, in which the infant dose was administered at either 3, 4, or 6 months of age, it was found that the earlier the infant dose was administered, the more additional cases, additional deaths, and additional cases with LTS were prevented in both the base-case IMD incidence scenario and the high IMD incidence scenarios. This suggests that the timing of the infant dose has a substantial effect on the protection afforded by the vaccination strategy and is consistent with the high incidence of IMD in infants relative to other age groups [1,5]. Furthermore, the strategy in which the infant MenACWY-TT dose was administered later (6 months) versus earlier (3 or 4 months) resulted in excess IMD cases, deaths, and cases with LTS under the high MenC scenario, due to the occurrence of MenC cases before the primary dose was administered (when primary vaccination occurred at 6 months there were an estimated 7489 cases; this dropped to 7379 and 7415 cases when the primary dose was administered at 3 and 4 months, respectively). A previous study in which a static multi-cohort population model was used to estimate the efficacy of three-dose MenACWY vaccination schedules (two infant doses + adolescent dose) in the United Kingdom also found that earlier infant dose strategies prevented the most IMD cases, deaths, and cases with LTS [26]. Together, these modeling studies suggest that for a MenACWY-TT dose schedule, administration of the vaccine in early infancy provides better protection.

Consistent with the findings from our model of increased protection against IMD with MenACWY-TT vaccination strategies, real-world analyses of vaccine effectiveness in the Netherlands, Australia, and the United Kingdom have shown that the introduction of MenACWY quadrivalent vaccines into NIPs in various age groups has been effective at reducing the incidence of IMD [22,23,33,34,35]. In the Netherlands, in 2018 MenACWY-TT was introduced into the NIP for toddlers 14 months of age, replacing MenC vaccination, and in 2020 it was also added for those 14 years of age [22]. In 2018 Australia’s NIP implemented MenACWY-TT for infants 12 months of age, replacing vaccination with MenC, and in 2019 for children 14 to 16 years of age [22]. Following the introduction of the single MenACWT-TT dose, an incidence rate reduction of 85% in the Netherlands and 83% in Australia for MenC/W/Y disease in toddlers and adolescents/young adults was observed [22]. In 2015 the NIP in England replaced vaccination with a single dose of an MenC vaccine in adolescents 13 to 14 years of age with an MenACWY vaccine, which resulted in a incidence rate reduction of 89%, 65%, and 79% for MenC, MenW, and MenY disease, respectively, among those 14 to 18 years of age [22]. Adolescents are important reservoirs of meningococcus, with carriage rates peaking at around 19 years of age; MenACWY vaccination strategies that reduce carriage in adolescents have been shown to interrupt onward transmission, conveying indirect protection against IMD to non-vaccinated age groups [35,36,37]. The static multi-cohort population model used here accounted for the indirect effects of vaccination (i.e., herd protection) using assumptions based on real-world observations following the introduction of MenACWY vaccination into national adolescent immunization programs [22]. Similarly, the model inputs for both the base-case and high-incidence scenarios were based on relevant, real-world epidemiological data, with the intention of generating realistic, applicable model estimates.

The model assumed MenACWY-TT vaccine effectiveness was the same for all four serogroups (MenA/C/W/Y), and that MenACWY-TT vaccine effectiveness after the primary vaccination was lower than that reported for the MenC-specific vaccine (MenACWY-TT: 85% or 89% with primary vaccination at 2/3/4 months or 6 months of age, respectively; MenC: 90% with primary vaccination at 4 months of age) [22,23,24,25]. Despite the assumption of a lower vaccine effectiveness after a single dose of MenACWY-TT versus MenC, the model predicted that the combined effects of direct and indirect protection against multiple serogroups with MenACWY-TT vaccination strategies offered greater protection against IMD than the monovalent MenC infant vaccination approach. Studies have shown that persistence of MenC antibodies after MenACWY-TT vaccination is greater or equivalent to persistence after vaccination with the MenC vaccine, suggesting that the vaccine effectiveness for MenACWY-TT used in our study was a conservative estimate [28,33,38,39,40,41]. On this basis, our modeled numbers of additional IMD cases, deaths, and cases with LTS prevented with the MenACWY-TT strategies may be an underestimation. It should be noted that the model used MenACWY-TT vaccine effectiveness inputs that were based on MenACWY-TT-specific data, and therefore may not be applicable to other quadrivalent MenACWY vaccines [22,23,24].

The model in the current manuscript showed that vaccination with MenACWY-TT at 2, 4, and 12 months of age with a fourth dose at 12 years of age had the greatest impact in terms of additional cases, deaths, and cases with LTS prevented. However, national decision-making around adoption of new immunization policies requires a holistic approach that, in addition to vaccine efficacy, needs to consider affordability, cost-effectiveness, and feasibility of implementation. In Spain, switching from a MenC dose to a MenACWY dose at 4 and 12 months of age offers the simplest and potentially most affordable option because no new additional clinic visits would be required. The vaccination strategies in which initial MenACWY doses are administered at 2 to 4 months of age would be convenient as many other vaccines are administered at this age and MenACWY could be co-administered with the other recommended vaccines [17]. A similar approach is also being taken in France where transition from MenC to MenACWY vaccination in infants was implemented from January 2025 [42]. A recent phase 3B study that evaluated immunogenicity responses to a ‘1 + 1’ infant MenACWY-TT vaccination schedule, with a primary dose administered at 3 months of age followed by a second dose at 12 months, reported seroprotective titers across MenA, MenC, MenW, and MenY serogroups in 82–91% of the 145 infants who received the primary dose, and 100% of the 143 infants who received a second dose [43]. These data provide support for the recommendations that have been adopted in Malta since 2020 for routine infant quadrivalent MenACWY vaccination at 3 and 13 months of age [44,45]. Based on the model estimates from the current study, real-world evidence evaluating MenACWY vaccine implementation in various countries, and MenACWY immunogenicity data from similar infant schedules, we anticipate that the introduction in Spain of a MenACWY-TT schedule of vaccination at 4 and 12 months of age with a third dose at 12 years of age will achieve similar success in increasing protection against multiple meningococcal serogroups.

Our model did not consider MenA disease because disease from serogroup A is rare in Spain; there were no cases of MenA in Spain in 2021, 2022, or 2023, and 1 case in 2024 [14,46]. However, should MenA be introduced into Spain from other regions, offering broad protection against all four serogroups with an MenACWY vaccine would protect infants and young children against MenA disease. Continuing to vaccinate adolescents with MenACWY vaccine would also be able to reduce carriage and transmission.

### Limitations

The mathematical models developed in this study, as with all models, are simplifications of real-world conditions, and there is some uncertainty in projecting long-term changes in unpredictable and continually evolving diseases, such as IMD. Model inputs rely on the robustness of available data to populate the model. Model inputs specific for Spain were not always available and estimates from other countries were sometimes applied. Additionally, incidence rates used in the base-case analysis may underestimate the burden of IMD in any given period; however, we did analyze effects of the different vaccination strategies in hypothetical high-incidence scenarios for MenC, MenW, and MenY. Assumptions used to calculate the herd effect were simplified for inclusion in the model population, although the assumptions were generally conservative; in addition, vaccine effectiveness estimates assumed that efficacy in a Spanish population would reflect the published MenABCWY-TT and MenC effectiveness data on which model inputs were based [22,23,24,25]. Finally, MenB, and the effects of vaccination against IMD caused by MenB, were not included in the model; this is important given the high prevalence of MenB in Spain and Europe relative to other serogroups, and evidence that recombinant vaccines that protect against MenB may also provide a degree of protection against IMD caused by non-MenB serogroups [1,3,14,47,48]. MenB vaccination has been included in infant/adolescent vaccination programs globally, and in Spain since 2023 [49], with an estimated effectiveness in Spain of 71% against MenB disease and 76% against any serogroup in fully vaccinated children < 5 years of age [50].

## 5. Conclusions

The static multi-cohort population model used in this study estimated that IMD cases, IMD-related deaths, and cases with LTS could be substantially lowered in Spain with the adoption of the MenACWY-TT vaccine in infants. These data support the decision made by several of the regional governments of Spain to adopt the updated vaccine recommendations of the Advisory Committee on Vaccines of the Spanish Association of Pediatrics, which aim to increase protection against IMD by transitioning from the MenC vaccine to the MenACWY-TT vaccine at 4 and 12 months of age, while maintaining MenACWY vaccination at 12 years of age. Even in the high MenC/W/Y incidence scenarios, model estimates demonstrate that adoption of MenACWY-TT in the infant vaccination schedule in Spain would better prepare the population against evolutionary changes in IMD, especially against disease caused by MenW and MenY. The robust model used in the current study, with inputs derived from real-world data, was designed to ensure realistic outputs that may be applicable in settings beyond Spain where monovalent MenC vaccination strategies are still relied upon.

## Figures and Tables

**Table 1:** IMD per 100,000 base-case incidence and modeled high-incidence rates by age range and per serogroup.

	Base-Case Incidence Rate			High Serogroup Incidence Scenario		
Age Range	MenC 1999–2006	MenW 2016–2019	MenY 2016–2019	High MenC 1999–2000	High MenW 2018–2019	High MenY 2018–2019
0–<3 mo	2.57	0.40	0.11	5.14	0.60	0.23
3–<12 mo	5.14	0.80	0.23	10.29	1.19	0.45
1–<2 y	6.04	0.23	0.08	12.03	0.36	0.08
2–<3 y	4.95	0.17	0.06	9.76	0.28	0.07
3–<4 y	3.85	0.12	0.05	7.49	0.20	0.05
4–<5 y	2.76	0.07	0.04	5.21	0.11	0.04
5–<15 y	1.06	0.04	0.07	1.86	0.07	0.08
15–<25 y	0.67	0.14	0.10	1.10	0.20	0.09
25–<50 y	0.10	0.03	0.02	0.19	0.05	0.03
50–<65 y	0.10	0.08	0.04	0.19	0.14	0.06
65–<100 y	0.14	0.23	0.17	0.26	0.34	0.27

IMD, invasive meningococcal disease; MenC, meningococcal serogroup C; MenW, meningococcal serogroup W; MenY, meningococcal serogroup Y.

**Table 2:** Potential IMD vaccination strategies modeled with MenC and/or MenACWY-TT vaccine and percentage vaccine effectiveness assumptions.

	Age at Vaccination					
Vaccination Strategy	2 mo	3 mo	4 mo	6 mo	12 mo	12 y
Reference strategy						
MenC vaccine at 4 and 12 mo + MenACWY-TT at 12 y			C 90%		C 92%	ACWY 94%
MenACWY-TT strategies						
MenACWY-TT at 2, 4, 12 mo + 12 y	ACWY 85%		ACWY 92%		ACWY 92%	ACWY 94%
MenACWY-TT at 4 and 12 mo + 12 y			ACWY 85%		ACWY 92%	ACWY 94%
MenACWY-TT at 3 and 12 mo + 12 y		ACWY 85%			ACWY 92%	ACWY 94%
MenACWY-TT at 6 and 12 mo + 12 y				ACWY 89%	ACWY 92%	ACWY 94%

IMD, invasive meningococcal disease; MenACWY-TT, meningococcal serogroup A, C, W, and Y tetanus toxoid conjugate vaccine; MenC, meningococcal serogroup C. Effectiveness assumptions are shown as percentages in each cell.

**Table 3:** Public health impact of various MenACWY-TT vaccination strategies compared with the reference vaccination strategy on IMD due to MenC/W/Y.

Vaccination Dosing Strategies	Cases	Deaths	Cases with LTS
Reference strategy			
MenC vaccine at 4 and 12 mo + MenACWY-TT at 12 y	5273	833	2124
MenACWY-TT strategies	Add’l cases prevented *	Add’l deaths prevented *	Add’l cases with LTS prevented *
MenACWY-TT at 2, 4, and 12 mo + 12 y	103	17	41
MenACWY-TT at 4 and 12 mo + 12 y	60	11	24
MenACWY-TT at 3 and 12 mo + 12 y	80	14	32
MenACWY-TT at 6 and 12 mo + 12 y	20	4	7

Add’l, additional; IMD, invasive meningococcal disease; LTS, long-term sequela; MenACWY-TT, meningococcal serogroup A, C, W, and Y tetanus toxoid conjugate vaccine; MenC, meningococcal serogroup C; MenC/W/Y, meningococcal serogroup C, W, and Y. * Add’l cases and deaths prevented are cases and deaths prevented relative to the cases and deaths that occurred with the reference vaccination strategy.

**Table 4:** Analysis of the impact of various vaccination strategies on MenC/W/Y cases and deaths in high MenC/W/Y incidence scenarios compared with the reference strategy.

High-Incidence Scenario *	Reference Strategy	MenACWY-TT Strategies			
	MenC Vaccine at 4 and 12 mo + MenACWY-TT at 12 y	At 2, 4, and 12 mo + 12 y	At 4 and 12 mo + 12 y	At 3 and 12 mo + 12 y	At 6 and 12 mo + 12 y
High MenC					
Cases	7465	7337	7415	7379	7489
Deaths	1164	1143	1155	1149	1167
Cases with LTS	3014	2963	2995	2980	3025
Additional cases prevented	—	128	50	87	−24
Additional deaths prevented	—	21	9	15	−2
Additional cases with LTS prevented	—	51	20	34	−11
High MenW					
Cases	6156	6023	6068	6047	6112
Deaths	1008	985	992	988	999
Cases with LTS	2463	2410	2429	2420	2446
Additional cases prevented	—	133	88	109	45
Additional deaths prevented	—	23	16	20	9
Additional cases with LTS prevented	—	53	34	43	17
High MenY					
Cases	5854	5742	5786	5766	5828
Deaths	900	882	889	886	895
Cases with LTS	2370	2325	2343	2335	2360
Additional cases prevented	—	112	68	89	26
Additional deaths prevented	—	18	12	15	5
Additional cases with LTS prevented	—	45	27	35	10
High MenC/W/Y					
Cases	8930	8762	8844	8806	8923
Deaths	1407	1379	1391	1385	1403
Cases with LTS	3599	3532	3566	3550	3597
Additional cases prevented	—	168	86	124	7
Additional deaths prevented	—	28	16	22	3
Additional cases with LTS prevented	—	67	34	49	2

LTS, long-term sequela; MenACWY-TT, meningococcal serogroup A, C, W, and Y tetanus toxoid conjugate vaccine; MenC, meningococcal serogroup C; MenC/W/Y, meningococcal serogroup C, W, and Y; MenW, meningococcal serogroup W; MenY, meningococcal serogroup Y. * Incidence is increased for high serogroups; other serogroups are kept at base-case incidence values.

## Data Availability

Upon request, and subject to review, Pfizer will provide the data that support the findings of this study.

## References

[1] Parikh S.R., Campbell H., Bettinger J.A., Harrison L.H., Marshall H.S., Martinon-Torres F., Safadi M.A., Shao Z., Zhu B., von Gottberg A. (2020). The everchanging epidemiology of meningococcal disease worldwide and the potential for prevention through vaccination. J. Infect..

[2] Wang B., Santoreneos R., Giles L., Haji Ali Afzali H., Marshall H. (2019). Case fatality rates of invasive meningococcal disease by serogroup and age: A systematic review and meta-analysis. Vaccine.

[3] Pardo de Santayana C., Tin Tin Htar M., Findlow J., Balmer P. (2023). Epidemiology of invasive meningococcal disease worldwide from 2010–2019: A literature review. Epidemiol. Infect..

[4] Olbrich K.J., Muller D., Schumacher S., Beck E., Meszaros K., Koerber F. (2018). Systematic review of invasive meningococcal disease: Sequelae and quality of life impact on patients and their caregivers. Infect. Dis. Ther..

[5] Acevedo R., Bai X., Borrow R., Caugant D.A., Carlos J., Ceyhan M., Christensen H., Climent Y., De Wals P., Dinleyici E.C. (2019). The Global Meningococcal Initiative meeting on prevention of meningococcal disease worldwide: Epidemiology, surveillance, hypervirulent strains, antibiotic resistance and high-risk populations. Expert Rev. Vaccines.

[6] Nuttens C., Findlow J., Balmer P., Swerdlow D.L., Tin Tin Htar M. (2022). Evolution of invasive meningococcal disease epidemiology in Europe, 2008 to 2017. Eurosurveillance.

[7] Stinson C., Burman C., Presa J., Abalos M. (2020). Atypical presentation of invasive meningococcal disease caused by serogroup W meningococci. Epidemiol. Infect..

[8] Campbell H., Andrews N., Parikh S., Ribeiro S., Gray S., Lucidarme J., Ramsay M.E., Borrow R., Ladhani S.N. (2020). Variable clinical presentation by the main capsular groups causing invasive meningococcal disease in England. J. Infect..

[9] Soneira M.S., Alférez M.D.R.C., Portero R.C. (2021). Meningococcal Disease. Seasons 2018–2019, 2019–2020.

[10] Soneira M.S., Alférez M.D.R.C., Portero R.C. (2022). Meningococcal Disease. 2020–2021 Season.

[11] Brueggemann A.B., Jansen van Rensburg M.J., Shaw D., McCarthy N.D., Jolley K.A., Maiden M.C.J., van der Linden M.P.G., Amin-Chowdhury Z., Bennett D.E., Borrow R. (2021). Changes in the incidence of invasive disease due to *Streptococcus pneumoniae*, *Haemophilus influenzae*, and *Neisseria meningitidis* during the COVID-19 pandemic in 26 countries and territories in the Invasive Respiratory Infection Surveillance Initiative: A prospective analysis of surveillance data. Lancet Digit. Health.

[12] Shen S., Findlow J., Peyrani P. (2024). Global epidemiology of meningococcal disease-causing serogroups before and after the COVID-19 pandemic: A narrative review. Infect. Dis. Ther..

[13] Moraga-Llop F., Andradas E., Blesa-Baviera L.C., Canton R., Gonzalez Del Castillo J., Martinon-Torres F., Moya E., Trilla A., Vazquez J., Villena R.J. (2024). Meningococcal meningitis in Spain in the Horizon 2030: A position paper. Rev. Esp. Quimioter..

[14] National Center for Epidemiology Instituto de Salud Carlos III (ISCIII). [Weekly Online Newsletter. Number 52. Year 2024]. https://cne.isciii.es/documents/d/cne/is_n-52-20241226_web.

[15] Tzanakaki G., Cabrnochova H., Delic S., Draganescu A., Hilfanova A., Onozo B., Pokorn M., Skoczynska A., Tesovic G. (2024). Invasive meningococcal disease in South-Eastern European countries: Do we need to revise vaccination strategies?. Hum. Vaccines Immunother..

[16] Clark S.A., Borrow R. (2020). Herd protection against meningococcal disease through vaccination. Microorganisms.

[17] Ministry of Health Spain Vaccines and Vaccination Program. https://www.sanidad.gob.es/areas/promocionPrevencion/vacunaciones/calendario/home.htm.

[18] Alvarez Garcia F.J., Iofrio de Arce A., Alvarez Aldean J., Garces-Sanchez M., Garrote Llanos E., Montesdeoca Melian A., Navarro Gomez M., Pineda Solas V., Rivero Calle I., Ruiz-Contreras J. (2024). Immunisation schedule of the Spanish Association of Pediatrics: 2024 recommendations. An. Pediatr. (Engl. Ed.).

[19] Xunta de Galicia Dirección Xeral de Saúde Pública Calendario de Inmunización ao Longo de Toda a Vida. https://www.sergas.es/Saude-publica/Calendario-comun-vacinacion.

[20] Junta de Andalucía Consejería de Salud y Consumo Vaccination Schedule Andalusia. https://www.andavac.es/calendario-vacunaciones/.

[21] Junta de Castilla y León Consejería de Sanidad Nuevo Calendario de Vacunaciones de Inmunizaciones Para Toda la Vida- Castilla y León 2024. https://www.saludcastillayleon.es/profesionales/es/vacunaciones/nuevo-calendario-vacunaciones-inmunizaciones-toda-vida-cast.

[22] Villena R., Kriz P., Tin Tin Htar M., Burman C., Findlow J., Balmer P., Jodar L. (2023). Real-world impact and effectiveness of MenACWY-TT. Hum. Vaccines Immunother..

[23] Ohm M., Hahné S.J.M., van der Ende A., Sanders E.A.M., Berbers G.A.M., Ruijs W.L.M., van Sorge N.M., de Melker H.E., Knol M.J. (2022). Vaccine impact and effectiveness of meningococcal serogroup ACWY conjugate vaccine implementation in the Netherlands: A nationwide surveillance study. Clin. Infect. Dis..

[24] European Medicines Agency Nimenrix Meningococcal Groups A, C, W-135 and Y Conjugate Vaccine. https://www.ema.europa.eu/en/medicines/human/EPAR/nimenrix.

[25] (2015). NeisVac-C Vaccine (Meningococcal Group C-TT Conjugate Vaccine, Adsorbed).

[26] Schley K., Kowalik J.C., Sullivan S.M., Vyse A., Czudek C., Tichy E., Findlow J. (2023). Assessing the role of infant and toddler MenACWY immunisation in the UK: Does the adolescent MenACWY programme provide sufficient protection?. Vaccines.

[27] Borja-Tabora C.F.C., Peyrani P., Webber C., Van der Wielen M., Cheuvart B., De Schrevel N., Bianco V., Aris E., Cutler M., Li P. (2020). A phase 2b/3b MenACWY-TT study of long-term antibody persistence after primary vaccination and immunogenicity and safety of a booster dose in individuals aged 11 through 55 years. BMC Infect. Dis..

[28] Vesikari T., Peyrani P., Webber C., Van Der Wielen M., Cheuvart B., De Schrevel N., Aris E., Cutler M., Li P., Perez J.L. (2020). Ten-year antibody persistence and booster response to MenACWY-TT vaccine after primary vaccination at 1–10 years of age. Hum. Vaccines Immunother..

[29] European Medicine Agency Nimenrix. Annex I: Summary of Product Characteristics. Last updated 16 December 2024. https://www.ema.europa.eu/en/medicines/human/EPAR/nimenrix#product-info.

[30] European Centre for Disease Prevention and Control Surveillance Atlas of Infectious Diseases. https://atlas.ecdc.europa.eu/public/index.aspx.

[31] Spanish Ministry of Health Vaccines and Vaccination Program. https://www.sanidad.gob.es/areas/promocionPrevencion/vacunaciones/coberturas/docs/Todas_las_tablas2020.pdf.

[32] Advisory Committee on Vaccines and Immunisations of the Spanish Association of Paediatrics Coberturas de Vacunación Frente a Meningitis C y Meninigitis ACWY en Adolescentes. Comunidades Autónomas. Año 2020. https://vacunasaep.org/sites/vacunasaep.org/files/minsanidad_coberturas-vacunales-2020-tabla07.pdf.

[33] Burman C., Knuf M., Safadi M.A.P., Findlow J. (2024). Antibody persistence and revaccination recommendations of MenACWY-TT: A review of clinical studies assessing antibody persistence up to 10 years after vaccination. Expert Rev. Vaccines.

[34] Campbell H., Edelstein M., Andrews N., Borrow R., Ramsay M., Ladhani S. (2017). Emergency meningococcal ACWY vaccination program for teenagers to control group W meningococcal disease, England, 2015–2016. Emerg. Infect. Dis..

[35] Campbell H., Andrews N., Parikh S.R., White J., Edelstein M., Bai X., Lucidarme J., Borrow R., Ramsay M.E., Ladhani S.N. (2022). Impact of an adolescent meningococcal ACWY immunisation programme to control a national outbreak of group W meningococcal disease in England: A national surveillance and modelling study. Lancet Child Adolesc. Health.

[36] Christensen H., May M., Bowen L., Hickman M., Trotter C.L. (2010). Meningococcal carriage by age: A systematic review and meta-analysis. Lancet Infect. Dis..

[37] Carr J.P., MacLennan J.M., Plested E., Bratcher H.B., Harrison O.B., Aley P.K., Bray J.E., Camara S., Rodrigues C.M.C., Davis K. (2022). Impact of meningococcal ACWY conjugate vaccines on pharyngeal carriage in adolescents: Evidence for herd protection from the UK MenACWY programme. Clin. Microbiol. Infect..

[38] Vesikari T., Forsten A., Boutriau D., Bianco V., Van der Wielen M., Miller J.M. (2012). Randomized trial to assess the immunogenicity, safety and antibody persistence up to three years after a single dose of a tetravalent meningococcal serogroups A, C, W-135 and Y tetanus toxoid conjugate vaccine in toddlers. Hum. Vaccines Immunother..

[39] Knuf M., Baine Y., Bianco V., Boutriau D., Miller J.M. (2012). Antibody persistence and immune memory 15 months after priming with an investigational tetravalent meningococcal tetanus toxoid conjugate vaccine (MenACWY-TT) in toddlers and young children. Hum. Vaccines Immunother..

[40] Vesikari T., Forsten A., Bianco V., Van der Wielen M., Miller J.M. (2015). Immunogenicity, safety and antibody persistence of a booster dose of quadrivalent meningococcal ACWY-tetanus toxoid conjugate vaccine compared with monovalent meningococcal serogroup C vaccine administered four years after primary vaccination using the same vaccines. Pediatr. Infect. Dis. J..

[41] Knuf M., Helm K., Kolhe D., Van Der Wielen M., Baine Y. (2018). Antibody persistence and booster response 68 months after vaccination at 2–10 years of age with one dose of MenACWY-TT conjugate vaccine. Vaccine.

[42] French Ministry of Labor, Health and Solidarity [The Vaccination Schedule]. https://sante.gouv.fr/prevention-en-sante/preserver-sa-sante/vaccination/calendrier-vaccinal?lang=en.

[43] Koski S., Martinon-Torres F., Rämet M., Zolotas L., Newton R., Maansson R., Cuter M.P.P., Findlow J., Balmer P., Jodar L. Meningitis Research Foundation Conference 2023 Poster Abstract Book. #P21 A Phase 3B, Open-Label Study to Evaluate the Safety and Immunogenicity of MenACWY-TT Vaccine in Healthy Infants Given at 3 and 12 Months of Age. https://www.meningitis.org/getmedia/41dcca45-856d-45cc-97f1-00465ea928c6/MRF-Conference-2023-Poster-Abstract-Book_v2.

[44] European Centre for Disease Prevention and Control Vaccine Scheduler. Vaccine Schedules in All Countries of the European Union. https://vaccine-schedule.ecdc.europa.eu/.

[45] Government of Malta Primary HealthCare. https://primaryhealthcare.gov.mt/en/.

[46] National Center for Epidemiology Instituto de Salud Carlos III (ISCIII). [Weekly Online Newsletter. Number 42. Year 2023]. https://cne.isciii.es/documents/d/cne/is_n-42-20231017_web-pdf.

[47] Ladhani S.N., Campbell H., Andrews N., Parikh S.R., White J., Edelstein M., Clark S.A., Lucidarme J., Borrow R., Ramsay M.E. (2021). First real-world evidence of meningococcal group B vaccine, 4CMenB, protection against meningococcal group W disease: Prospective Enhanced National Surveillance, England. Clin. Infect. Dis..

[48] Harris S.L., Tan C., Andrew L., Hao L., Liberator P.A., Absalon J., Anderson A.S., Jones T.R. (2018). The bivalent factor H binding protein meningococcal serogroup B vaccine elicits bactericidal antibodies against representative non-serogroup B meningococci. Vaccine.

[49] Abitbol V., Martinon-Torres F., Taha M.K., Nolan T., Muzzi A., Bambini S., Borrow R., Toneatto D., Serino L., Rappuoli R. (2024). 4CMenB journey to the 10-year anniversary and beyond. Hum. Vaccines Immunother..

[50] Castilla J., Garcia Cenoz M., Abad R., Sanchez-Cambronero L., Lorusso N., Izquierdo C., Canellas Llabres S., Roig J., Malvar A., Gonzalez Carril F. (2023). Effectiveness of a meningococcal group B vaccine (4CMenB) in children. N. Engl. J. Med..

